# Cardiac Hypertrophy: A Comprehensive Review from Prenatal Life to Young Adulthood

**DOI:** 10.3390/children12080989

**Published:** 2025-07-28

**Authors:** Martina Avesani, Elettra Pomiato, Sara Moscatelli, Jolanda Sabatino, Nunzia Borrelli, Leonie Luedke, Rosalba De Sarro, Sara Pavesi, Giulia Pelaia, Claudio Mastellone, Isabella Leo, Giovanni Di Salvo

**Affiliations:** 1 Paediatric Cardiology Unit, Department of Woman’s and Child’s Health, University Hospital of Padua, 35128 Padua, Italysara.pavesi@aopd.veneto.it (S.P.);; 2 Centre for Paediatric Inherited and Rare Cardiovascular Disease, Institute of Cardiovascular Sciences, University College London, London WC1E 6BT, UK; 3 Centre for Inherited Cardiovascular Diseases, Great Ormond Street Hospital, London WC1N 3JH, UK; 4 Department of Experimental and Clinical Medicine, Magna Graecia University, 88100 Catanzaro, Italyrosalba.desarro@studenti.unicz.it (R.D.S.);; 5 Adult Congenital Heart Disease and Congenital and Familial Arrhythmias Unit, Monaldi Hospital, University of Campania “Luigi Vanvitelli”, 80131 Naples, Italy; 6 Paediatric Unit, Department of Experimental and Clinical Medicine, Magna Graecia University, 88100 Catanzaro, Italy

**Keywords:** pediatric cardiology, hypertrophic cardiomyopathy, echocardiography

## Abstract

Myocardial hypertrophy (MH) represents a complex and heterogeneous condition in the pediatric and young adult population. While rare in children, MH encompasses a wide spectrum of physiological and pathological entities, ranging from transient hypertrophy in the infants of diabetic mothers to progressive genetic hypertrophic cardiomyopathies (HCM) with significant morbidity and mortality. Differential diagnosis is critical, as many phenocopies—including metabolic, mitochondrial, and syndromic diseases—can mimic HCM. Echocardiography remains the first-line imaging modality, with cardiac magnetic resonance (CMR) and molecular diagnostics increasingly used for detailed characterization. Risk stratification tools, such as the HCM Risk-Kids model, support clinical decision-making but must be integrated with individualized assessment. Advances in prenatal screening and genetic testing have significantly improved outcomes, though long-term management requires multidisciplinary care. Understanding age-specific presentations and the underlying etiologies is essential for accurate diagnosis and targeted treatment. This review provides a comprehensive overview of cardiac hypertrophy from fetal life through young adulthood, with a focus on etiologies, diagnostic approaches, imaging modalities, and therapeutic strategies, and aims to guide clinicians through the evolving landscape of MH, emphasizing early recognition, comprehensive evaluation, and personalized care.

## 1. Introduction

Myocardial hypertrophy (MH), defined as an increase in left ventricular (LV) wall thickness greater than two standard deviations above the predicted mean corrected for body surface area (Z-score ≥ 2.0) [1], is a rare condition in pediatric age, encompassing a broad array of clinical conditions. Usually diagnosed with echocardiography, MH can be either the result of a physiological response to increased hemodynamic overload or represent a pathological manifestation of diverse genetic, metabolic, structural, or hemodynamic abnormalities [2,3,4].

The clinical trajectory of pediatric MH differs markedly from that observed in adults. Children might be at an increased risk for major adverse cardiac events, including arrhythmias and sudden cardiac death, with some events occurring early after diagnosis [5]. Nonetheless, survival rates have improved due to advances in diagnosis and therapy, with recent studies reporting 5- and 10-year survival exceeding 90%. Prognosis is significantly influenced by the age at onset, the underlying etiology, and the presence of risk factors, such as left ventricle outflow tract obstruction (LVOTO), family history of sudden cardiac death (SCD), and arrhythmias [6]. Current guidelines encourage the use of age-adapted diagnostic and risk stratification tools, reflecting the need to differentiate between benign, transient hypertrophy and progressive, high-risk forms [7]. Advanced imaging techniques and molecular diagnostics are key in this process and have improved the ability to define the underlying etiologies and risk profiles, guiding therapeutic management.

This review paper aims to provide a comprehensive overview of MH from fetus to young adults, examining the etiologies, diagnostic pathways, and prognostic implications.

## 2. Fetal Myocardial Hypertrophy

Fetal MH is defined as an increase in the thickness of the fetal heart walls exceeding two standard deviations above the mean for the gestational age [8]. It can be attributed to multiple factors, including maternal health conditions, fetal disorders, and metabolic and genetic issues. However, many causes of MH remain “idiopathic” [9,10].

One of the most well-established maternal contributors to this condition is diabetes, both in its gestational and pregestational form (Type 1), even in patients with good glycemic control, although recent data suggest that the risk of fetal MH is higher in fetuses of mothers with higher HbA1c [11]. The effects of maternal diabetes on fetal heart can be observed as early as the first trimester and tend to become more pronounced in the third trimester [12]. The hypertrophy is usually asymmetrical, and the interventricular septum is mostly, but not exclusively, involved. However, this condition is considered benign, as complete hypertrophy regression is generally observed between 6 and 12 months of life [13,14].

The ingestion of nonsteroidal anti-inflammatory drugs, herbal teas, and foods rich in polyphenols during pregnancy has also been associated with premature constriction/closure of the ductus arteriosus [15]. This condition, which occurs rarely before 27 weeks of gestation, causes a sudden increase in right ventricular (RV) afterload, resulting in RV hypertrophy, dilation, and dysfunction, with a wide range of clinical outcomes if not recognized and treated [16].

Among the fetal causes of MH, twin–twin transfusion syndrome (TTTS) should also be mentioned. This condition affects approximately 10–15% of monochorionic pregnancies and is associated with high fetal mortality and morbidity [17]. TTTS is characterized by an unbalanced flow across the placental vascular anastomoses, causing the transfer of volume and vasoactive substances from one twin (donor) to the other (recipient), who can develop cardiac enlargement, biventricular hypertrophy, systolic and diastolic dysfunction, and, ultimately, fetal hydrops.

Structural congenital heart defects represent yet another significant cause of fetal MH. Left ventricular hypertrophy (LVH) is most frequently caused by aortic valve stenosis as a consequence of increased afterload. Importantly, the stenosis can progress through pregnancy, causing LV dilatation and dysfunction. In some cases, it can result, ultimately, in hypoplastic left heart syndrome [18]. RV hypertrophy can be caused by pulmonary stenosis, especially in the most severe forms, and pulmonary atresia with an intact ventricular septum. In this condition, the morphological presentation of the right ventricle may vary, ranging from a severely hypoplastic chamber with marked hypertrophy of the RV myocardium to a dilated right ventricle with functional impairment [19]. Of note, the presence of fetal MH of unknown cause, whether or not associated with congenital heart defects and extracardiac findings, should raise suspicion of an underlying RASopathy [20].

Cardiac tumors, particularly rhabdomyomas, may present with imaging features that closely resemble MH. These tumors are most frequently associated with tuberous sclerosis complex, a genetic disorder caused by mutations in the TSC1 and TSC2 genes [21].

In addition, congenital disorders of glycosylation, mitochondrial respiratory chain deficiency, anemia, and chromosomal abnormalities (i.e., Trisomy 13) have also been linked to MH, but a systemic involvement is usually present in these conditions [9,10,22,23]. Lastly, anecdotal reports of familial hypertrophic cardiomyopathies have been published as well [9,10].

The identification and assessment of fetal MH involve collaboration between specialists, including gynecologists, fetal/pediatric cardiologists, and geneticists. Fetal echocardiography serves as the primary diagnostic tool. It is typically performed between the 18th and 22nd weeks of gestation, although earlier exams can be performed as early as between 12 and 14 weeks of gestation in fetuses at high risk for cardiac disorders. If there is a concern for evolving MH, ongoing surveillance with serial evaluations at intervals of 2 to 4 weeks is usually advised for up to 34 to 36 weeks’ gestation [8].

**Figure 1:**
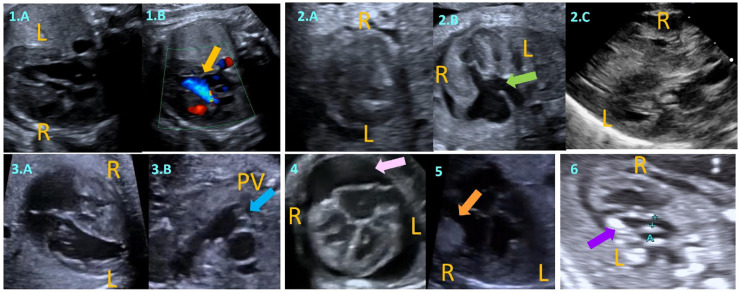
(**1.A**,**1.B**): Symmetric biventricular hypertrophy with left ventricular outflow tract obstruction (LVOTO), suggestive of RASopathy; color Doppler in (**1.B**) highlights turbulent flow (yellow arrow); (**2.A**–**2.C**): Progressive myocardial hypertrophy in a fetus of a diabetic mother with complete atrioventricular septal defect (cAVSD), likely with an associated metabolic disorder. (**2.A**,**2.B**) Fetal progression across gestation and (**2.C**) postnatal persistence. Green arrow in (**2.B**) indicates ostium primum defect; (**3.A**,**3.B**): right ventricular hypertrophy due to pulmonary stenosis. The pulmonary valve (PV) is indicated by a blue arrow in (**3.B**); (**4**): marked biventricular hypertrophy with associated pericardial effusion (pink arrow), suggesting hydrops or cardiac compromise; (**5**): echogenic intracardiac mass (orange arrow) consistent with rhabdomyoma, mimicking hypertrophy; (**6**): hypoplastic left heart syndrome (HLHS) with echogenic endocardium (purple arrow), consistent with endocardial fibroelastosis. L: Left; R: Right.

Prenatal ultrasound imaging, illustrating various patterns and etiologies of fetal myocardial hypertrophy, is shown in Figure 1.

Prenatal counseling plays a fundamental role, especially when genetic factors are suspected to contribute to MH. While in certain cases, particularly those related to maternal diabetes, hypertrophy may regress following birth, in cases where structural, genetic, or metabolic factors are involved, the condition might follow a more progressive course, necessitating careful monitoring, long-term follow-up, and potential therapeutic interventions. For example, severe hypertrophy may impair ventricular function, increasing the risk of neonatal heart failure (HF), and structural congenital heart defects can require immediate postnatal surgical intervention [8].

Genetic tests can be offered in specific settings, according to the International Society of Ultrasound in Obstetrics and Gynecology [24]. In families with a history of inherited HCM, prenatal or pre-implantation genetic diagnosis may be considered in the following cases: (1) a previous child with an inherited cardiomyopathy caused by one or more pathogenic genetic variants; (2) one or both prospective parents identified as carriers of a known familial pathogenic variant; (3) a family history marked by severe clinical manifestations of the disease in a parent or close relative [7]. However, according to some experienced centers, it is not appropriate to carry out these tests because of the significant variability in disease severity, age of onset, and phenotypic expression, which greatly limit the ability to predict the clinical course. This information should be clearly communicated to the couple during the pre-test counseling for prenatal diagnosis [25].

## 3. Etiology, Diagnosis, and Risk Stratification of Ventricular Hypertrophy in Children and Adolescents

LVH is a heterogeneous myocardial abnormality characterized by an increase in left ventricular mass [26]. This structural change typically results from an increase in wall thickness, an enlargement of the left ventricular cavity, or both. While LVH in adults is commonly associated with systemic hypertension or aortic stenosis (AS), in children, the condition reflects a broader spectrum of underlying etiologies, often unrelated to loading conditions [26].

The most frequent cause of LVH in childhood is sarcomeric HCM, particularly in older children and adolescents. HCM is typically inherited in an autosomal dominant manner and results from pathogenic variants in genes encoding sarcomeric proteins, most commonly MYH7 and MYBPC3. However, in neonates and infants, LVH may instead reflect inborn errors of metabolism, mitochondrial disease, or syndromic conditions such as Noonan syndrome, which often have systemic involvement. This age-related variability in the etiology underscores the importance of a thorough diagnostic evaluation, including genetic testing and metabolic screening, where appropriate [26,27].

Childhood-onset cardiomyopathies are uncommon, with an annual incidence of 1.3 per 100,000, and remain poorly understood [27]. The pathophysiological mechanisms of LVH in pediatric patients require deeper investigation due to complex and age-dependent variations in clinical presentation and risk profile. From metabolic and syndromic disorders in neonates to sarcomeric cardiomyopathies and athletic adaptations in adolescents, LVH in children necessitates a stratified, precision-based diagnostic and therapeutic approach in order to ensure accurate diagnosis and prevent adverse outcomes, such as arrhythmias, progressive heart failure, and sudden cardiac death [26].

Etiological Spectrum Across Age Groups

The etiology of LVH in children varies markedly with age and is broader and more heterogeneous than in adult populations. A multicenter UK study of 687 pediatric patients with HCM found that 63% had non-syndromic HCM, most commonly due to pathogenic variants in sarcomeric genes, such as MYH7 (11.5%) and MYBPC3 (9%) [28]. However, the relative contribution of different etiologies shifts significantly across age groups.

Among infants (<1 year of age), RASopathies (primarily Noonan syndrome) accounted for 42%, inborn errors of metabolism (IEM) for 18.9%, and non-syndromic HCM for 28.3%. In contrast, in children aged 1–12 years, non-syndromic HCM represented the majority at 66.9%, with RASopathies contributing 11.2%, IEM 6.4%, and Friedreich’s ataxia around 8.6%. In adolescents (13–16 years), the predominance of non-syndromic HCM further increased, reaching over 75%, while RASopathies and metabolic disorders became increasingly rare [28,29,30].

Genetic testing revealed a pathogenic or likely pathogenic variant in 70.6% of infants tested, reinforcing the value of early molecular investigation. Data from the ESC EURObservational Research Programme (EORP) showed that rare disease phenocopies—which include syndromic and metabolic forms—were more common in patients under 10 years of age (30.9%) compared to those aged 10–18 years (19.6%) [29].

Family history was reported in 36% of patients, with 88% of those cases presenting with non-syndromic HCM. Interestingly, the median age at diagnosis for patients with a family history was 11 years, compared to 6 years for those without, highlighting how familial sarcomeric disease tends to manifest later in childhood [29].

Differential Diagnosis of Childhood LVH

While sarcomeric HCM remains the most frequent etiology in older children and adolescents, a comprehensive diagnostic approach must consider a range of phenocopies across all age groups. According to the 2023 ESC Guidelines, a “Red Flag” approach—integrating clinical phenotype, family history, systemic involvement, and targeted testing—is essential for distinguishing HCM from its mimics [7].

Syndromic conditions, such as Noonan syndrome (commonly due to PTPN11 and other RAS/MAPK variants), often present in infancy with short stature, facial dysmorphism, and complex valvular abnormalities alongside hypertrophy [28]. Metabolic disorders include Pompe disease (GAA pathogenic variants), characterized by biventricular hypertrophy, hepatosplenomegaly, and hypotonia, and Danon disease (LAMP2 pathogenic variant), presenting in males with concentric LVH, skeletal myopathy, and pre-excitation [29]. Mitochondrial disorders, such as ACAD9 deficiency, and glycogen storage diseases [e.g., debrancher enzyme or phosphorylase kinase B deficiency] may also manifest with hypertrophy and systemic signs. Neuromuscular diseases like Friedreich’s ataxia can mimic HCM in later childhood, with associated ataxia, areflexia, diabetes, and concentric LVH [29].

Imaging findings such as concentric or asymmetric hypertrophy, presence of LVOTO, or fibrosis patterns on cardiac MRI may support specific diagnoses. Biochemical and genetic testing are crucial for identifying the underlying etiologies and guiding personalized treatment, especially with emerging therapies, such as enzyme replacement for Pompe disease and MEK inhibitors in RASopathies [31,32].

Clinical Presentation and Risk Stratification of LVH in Childhood HCM

Risk stratification in childhood HCM has advanced considerably with the development of dedicated pediatric risk models, most notably the HCM Risk-Kids model. This model estimates the 5-year risk of sudden cardiac death (SCD) using a combination of non-invasive clinical variables, including maximal left ventricular wall thickness, non-sustained ventricular tachycardia (NSVT), unexplained syncope, and left atrial diameter [33]. In the largest external validation to date, a predicted 5-year risk threshold of ≥6% successfully identified over 70% of SCD events, confirming its predictive utility in a real-world setting [33].

Importantly, HCM Risk-Kids demonstrated good performance across diverse populations, including validation cohorts that were older and had fewer traditional risk markers, yet a comparable incidence of SCD. This suggests that the model remains robust despite phenotypic differences in disease expression [33]. However, similar to many risk tools, it performed best in low- to intermediate-risk patients and tended to underestimate the risk in the highest-risk individuals, reinforcing the importance of supplementing the model output with clinical judgment [33].

Compared to the PRIMaCY model, another recently developed pediatric risk calculator, HCM Risk-Kids, demonstrated superior calibration and discriminatory ability, as well as broader external validation [33,34]. Unlike adult risk models—such as the ESC HCM Risk-SCD tool—pediatric models place less weight on family history, acknowledging its limited predictive value in younger patients [35]. Overall, while HCM Risk-Kids is a significant advancement in pediatric HCM care, it should be used as a decision-support tool rather than a replacement for individualized assessment.

**Scheme 1:**
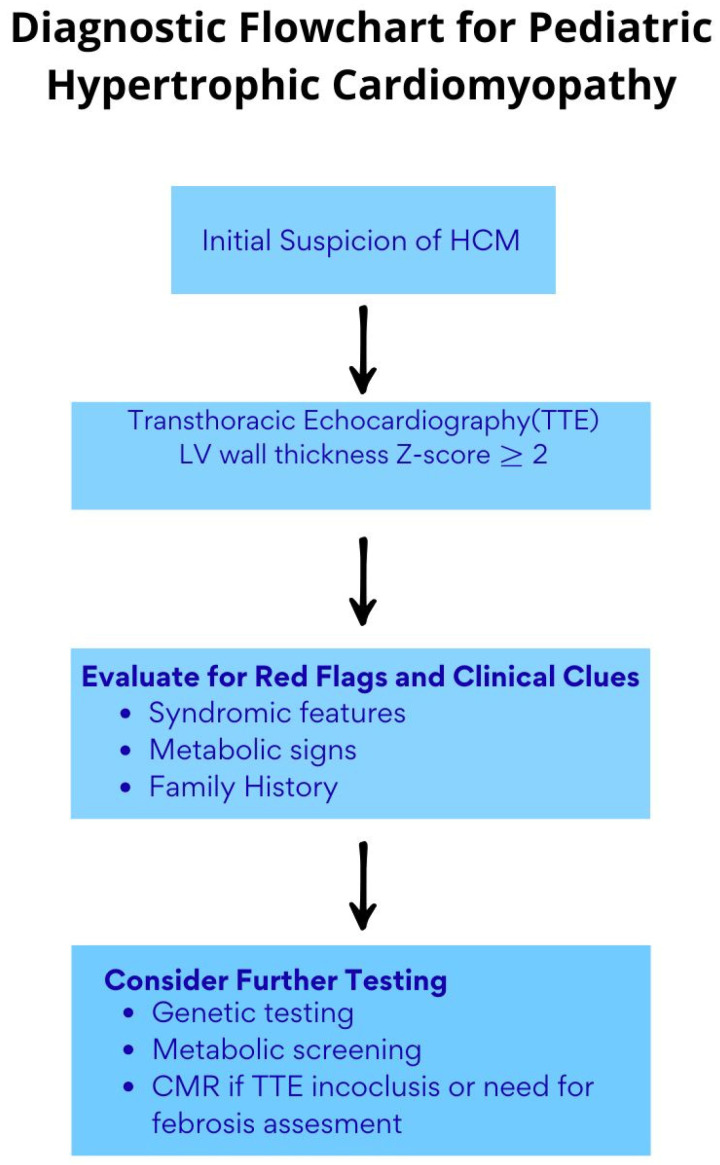
(HCM) hypertrophic cardiomyopathy; (CMR) cardiac magnetic resonance.

Scheme 1 summarizes the diagnostic approach to MH in young patients.

## 4. Imaging Assessment

- Echocardiography

Transthoracic echocardiography (TTE) represents the cornerstone imaging modality for both diagnosis and follow-up of MH in the pediatric and young adult population.

Its accessibility, non-invasiveness, and capacity to provide real-time assessment make it an essential tool, particularly in neonates and infants, where MH may be the first clinical manifestation of an underlying syndromic, metabolic, or infiltrative disorder [36], such as diabetes, glycogen storage diseases such as Pompe disease, and RASopathies such as Noonan syndrome [6,37,38].

From birth through adolescence, transthoracic echocardiography TTE allows for a comprehensive evaluation of myocardial wall thickness. Z-scores—adjusted for age and body surface area—are crucial, as they provide a standardized reference to identify myocardial hypertrophy [37] accurately.

However, MH in children represents a diagnostic and clinical challenge, particularly in the context of suspected HCM. While a left ventricular wall thickness z-score > 2 is often cited as a diagnostic threshold, it is critical to recognize that a z-score just under 2 SD does not exclude the diagnosis, especially in children with a familial predisposition to HCM or known pathogenic sarcomeric mutations [39,40]. Pediatric growth trajectories are dynamic and non-linear, and echocardiographic data are derived from heterogeneous populations, limiting strict cross-sectional application of z-scores across ages and ethnic groups [41]. Moreover, LVH in children may arise from a variety of hemodynamic stressors, including AS, coarctation of the aorta (CoA), and systemic hypertension [5]. In fact, the coexistence of HCM with congenital heart disease (CHD), such as inflow anomalies, is increasingly recognized, highlighting hypertrophy as a shared morphological hallmark rather than a disease-specific signature [42].

Echocardiography allows for the assessment of left ventricular geometry, enabling the distinction between concentric and eccentric hypertrophy and the detection of dynamic LVOTO. An assessment of the left ventricular systolic function and identification of associated valvular abnormalities, such as systolic anterior motion (SAM) of the mitral leaflet, complete the morpho-functional characterization required for diagnosis and follow-up planning [39].

The conventional emphasis on asymmetrical septal hypertrophy in adult HCM is often misleading in pediatric populations, where hypertrophy can present as severe, diffuse, or even symmetrical—features that should prompt suspicion for metabolic or mitochondrial cardiomyopathies [43,44,45]. Extreme forms of hypertrophy are not rare in children and may mimic infiltrative or storage disorders. Furthermore, structural abnormalities are frequently coexistent, including mitral valve leaflet elongation, anterior displacement or abnormal insertion of papillary muscles, and the presence of myocardial crypts—features that support a morphologically enriched HCM phenotype. Notably, pediatric patients with sarcomeric HCM may display overlapping features of other cardiomyopathic forms, such as dilated (DCM), restrictive (RCM), noncompacted myocardium (LVNC), or even RV hypertrophy, underscoring the importance of comprehensive phenotyping and genetic evaluation [46].

These parameters are essential for differentiating HCM from secondary or physiological hypertrophy, corroborating the diagnostic suspicion, and directing clinical management, including therapeutic strategies and potential indications for targeted genetic testing [47,48].

Advanced Echocardiography and Stress Echocardiography

Despite the central role of echocardiography in the assessment of diastolic function, conventional parameters (E/A ratio, E/E′, deceleration time, and indexed left atrial volume) show limited sensitivity in pediatric patients, particularly in the early or subclinical stages of disease, as demonstrated by Dragulescu et al. [49,50,51].

In this context, speckle-tracking echocardiography (STE) has emerged as a valuable diagnostic and prognostic tool in clinical practice.

Studies in children with HCM have demonstrated that STE can detect subclinical myocardial mechanical abnormalities not evident with conventional techniques. Dorobantu et al. reported significantly less negative global longitudinal strain (GLS) values in pediatric HCM patients compared to healthy controls, despite preserved systolic function, suggesting early subclinical myocardial involvement. Additionally, abnormalities in rotational mechanics, such as increased apical rotation and decreased basal rotation, have been described and may be linked to specific genetic profiles [52].

These findings are consistent with those of Forsey et al., who documented increased apical rotation in genotype-positive, phenotype-negative children, further supporting the role of STE in early detection of mechanical dysfunction even in the absence of overt structural changes [53].

Regarding atrial strain, Sabatino et al. demonstrated a significant reduction in left atrial strain in children with HCM, which was inversely correlated with invasively measured left ventricular end-diastolic pressure. These findings reinforce the value of atrial strain as a non-invasive and early marker of diastolic dysfunction in the pediatric population [54].

Finally, the echocardiographic method with stress TTE serves as a complementary tool in the functional evaluation of HCM. Although clinical experience in the pediatric population remains more limited compared to adults, recent studies [55,56] have confirmed its high technical feasibility and good tolerability. Stress echocardiography enables, particularly, the detection of dynamic LVOT gradients under stress in approximately one-third of pediatric HCM patients, which are not apparent at rest, thereby strengthening its diagnostic and follow-up utility [55,56].

Advanced Imaging

In complex cases or when TTE yields inconclusive results, second-line imaging modalities may be required. Among these, cardiac magnetic resonance imaging (CMR) is the most appropriate tool for assessing MH in pediatric patients [57,58].

CMR offers high reproducibility in quantifying ventricular volumes and myocardial wall thickness, as demonstrated by the multicenter study by Fogel et al. [59,60].

Due to its high spatial resolution, CMR is especially useful in detecting hypertrophy localized to the apical or lateral segments, areas where echocardiography may underestimate wall thickness, as well as the investigation of the right ventricle [58,61]. Beyond morphological assessment, CMR provides advanced tissue characterization through late gadolinium enhancement and T1 mapping techniques, which enable early identification of interstitial myocardial fibrosis, a recognized prognostic marker in HCM [58,62].

These techniques facilitate the distinction of hypertrophic patterns in different clinical contexts, including metabolic cardiomyopathies and genetic syndromes, thus improving diagnostic accuracy and supporting personalized treatment strategies [63,64]. In addition, it allows a deep assessment of the right ventricle.

A further valuable application is the ability of CMR to discriminate between physiologic athlete’s heart and HCM in adolescents, as the presence of asymmetric hypertrophy and myocardial fibrosis detectable via LGE makes it particularly useful in differential diagnosis [65,66].

Despite its diagnostic value, CMR still presents limitations in the pediatric setting. The extended time required for image acquisition and the breath-holding can be challenging in younger children, often requiring sedation or general anesthesia, which increases procedural risks. Moreover, the availability of CMR and the need for trained operators may limit its accessibility in some centers.

Finally, computed tomography (CT) and nuclear imaging may play complementary roles in selected pediatric scenarios. These modalities are particularly useful in cases requiring detailed anatomical evaluation of coronary arteries or the aorta, such as CoA or AS—conditions often associated with secondary hypertrophy. Cardiac CT, with its high spatial resolution and rapid acquisition, is preferred when CMR is contraindicated (incompatible device) or the radiation exposure needs to be minimized [67,68,69].

Nuclear imaging (SPECT or PET) can provide functional insights in cases of suspected ischemia or impaired coronary reserve, as in advanced HCM or secondary forms [70,71].

However, the use of ionizing radiation requires a careful risk–benefit assessment, especially in neonates and young children.

**Figure 2:**
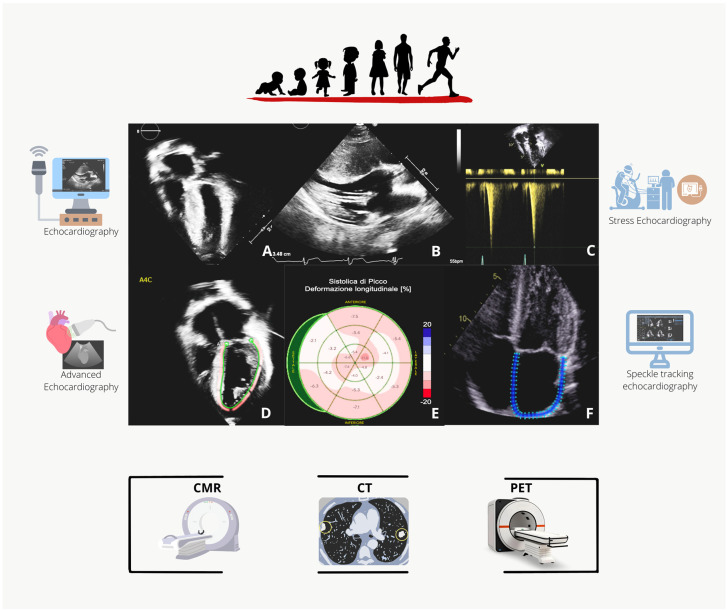
(**A**) Apical four-chamber view with end-systolic measurement of the left ventricular internal dimension; (**B**) Parasternal long-axis view showing marked hypertrophy of the interventricular septum; (**C**) Pulsed-wave Doppler interrogation across the left ventricular outflow tract under stress echocardiography conditions, to evaluate dynamic obstruction and exercise-induced gradients; (**D**) Apical four-chamber view with speckle tracking echocardiography; (**E**) Bull’s eye plot displaying peak systolic global longitudinal strain (GLS), with identification of regional strain reduction indicative of early systolic dysfunction; (**F**) Left atrial strain analysis derived from speckle tracking imaging. In addition to echocardiographic assessment, advanced cardiac imaging may be indicated in selected cases. (CMR) cardiac magnetic resonance; (CT) tomography; (PET) positron emission tomography.

Overall, the choice of imaging should always be guided by age, clinical picture, possibility of collaboration, and principles of appropriateness and safety [68]. The use of different imaging modalities to assess MH is summarized in Figure 2.

## 5. The Role of Genetics

Familial HCM is predominantly caused by mutations in sarcomeric protein genes, with autosomal dominant inheritance, and accounts for approximately 40–60% of all HCM cases. The most commonly affected genes include MYH7 (encoding beta-myosin heavy chain) and MYBPC3 (myosin-binding protein C), followed by TNNT2, TNNI3, MYL2, and MYL3 [7].

Less frequent genetic causes are linked to infiltrative or metabolic diseases, such as GLA mutations in Fabry disease, TTR in transthyretin amyloidosis, LAMP2 in Danon disease, and PRKAG2 in glycogen storage cardiomyopathy. These represent HCM phenocopies, which are disorders that resemble sarcomeric HCM in clinical characteristics but have different pathogenesis, natural histories, and treatment [72].

Genetic testing fulfills several functions: validating a molecular diagnosis, directing familial screening, and distinguishing sarcomeric HCM from phenocopies. However, only about 30% of patients meeting clinical criteria for HCM carry a pathogenic sarcomere gene mutation. Therefore, most cases are genetically unexplained, and a negative test does not exclude the disease [25].

Upon identification of a pathogenic variation in a proband, cascade testing of first-degree relatives can elucidate their risk status. Individuals who test negative for the familial mutation are unlikely to develop HCM and may typically be relieved from lifelong clinical monitoring. This is particularly relevant in asymptomatic carriers and helps reduce psychological burden and unnecessary follow-up [73].

Importantly, genetic test interpretation is complex. In 5–10% of families, initial classification of a variant may change over time due to evolving databases and clinical evidence, leading to potential misclassification and inappropriate management decisions. These uncertainties underscore the importance of expert genetic counseling [74].

According to the 2020 AHA/ACC guidelines, the majority of patients with HCM and their family members should undergo routine genetic testing. However, expert consensus expresses concerns about this approach, particularly considering its poor predictive value for outcomes like sudden cardiac death or disease progression [75].

Another relevant subset includes genotype-positive, phenotype-negative individuals—carriers of pathogenic variants without manifest disease. While they may transmit the mutation to offspring, most remain clinically stable, with low event rates and a low probability of phenotypic conversion in adulthood.

Targeted genetic testing is also essential for detecting non-sarcomeric conditions [74]. Distinguishing these diseases from sarcomeric HCM is essential, as they require distinct approaches to monitoring and treatment.

In children, genetic testing for HCM is commonly recommended starting around 10–12 years old [1,76] However, early screening should be considered in families with severe early-onset disease, in the presence of cardiac symptoms, or in those involved in high-intensity sports [77].

A promising approach to the future management of HCM is gene therapy, which aims to correct the underlying genetic defects to halt or prevent the development of the disease. Although still in its early stages, it has the potential to revolutionize care for certain high-risk groups [48].

## 6. Treatment Options

In patients with HCM, treatment should address four nodal points: LVOTO, atrial fibrillation (AF), signs and symptoms of HF, and prevention of SCD.

### 6.1. LVOTO

General measures

According to the most recent ESC Guidelines on cardiomyopathies, it is recommended that patients with obstructive HCM avoid dehydration and binge or significant alcohol consumption, especially older patients. Achievement and maintenance of normal BMI should also be promoted [7]. On the other hand, medications that increase the intraventricular gradient, such as vasodilators, nitrates, and phosphodiesterase inhibitors, as well as digoxin, should be avoided in patients with LVOTO. In case AF is present, rhythm or rate control should be achieved before taking into consideration invasive septal reduction therapies [78]. As the obstruction is volume-dependent, diuretics can be used at the minimal effective dose to alleviate dyspnea.

First-line therapy

In patients with spontaneous or triggered LVOT gradient ≥ 50 mmHg, different pharmacological or interventional strategies can be implemented, non-vasodilating beta-blockers being the recommended first-line therapy in symptomatic patients in both European and American guidelines [7], but they may also be considered in asymptomatic patients to decrease LV pressures, although the recommendation is less strong (IIb, level C) and based on expert consensus [7,79,80]. Beta-blockers reduce the heart rate, thus allowing adequate ventricular filling and ultimately reducing the exercise-induced LVOTO. Interestingly, data comparing different beta-blockers are scarce, although Dybro recently published a small double-blinded, placebo-controlled, randomized study demonstrating the efficacy of metoprolol in the symptoms, quality of life, and LVOT gradient in 29 patients [80].

However, beta-blockers can come with side effects, which include bradycardia, hypotension, fatigue, and different degrees of AV nodal block [81]. In case patients remain symptomatic despite the beta-blocker or in case of intolerance or contraindications to beta-blockers, non-dihydropyridine calcium channel blockers, namely verapamil or diltiazem, can be started and titrated to the maximum tolerated dose in symptomatic patients with LVOTO. The efficacy of these drugs has been established both in children and adults since the 1980s [75,82,83], and both are recommended by both the European and American guidelines (IB) [7,75]. Similar to beta-blockers, verapamil may also be considered in asymptomatic patients with LVOTO [81].

Second-line therapy

Traditionally, disopyramide, titrated to the maximum tolerated dose, is the recommended drug in addition to a beta-blocker (or to verapamil or diltiazem) in patients with LVOTO who remain symptomatic despite the initial treatment, as it has been shown to significantly reduce rest LVOT gradient and improve exercise capacity [7]. Although disopyramide is classified as a class Ia antiarrhythmic drug (ADD), clinical trials on HCM have shown it to have only a mild proarrhythmic effect and no associated increase in the risk of sudden cardiac death. Its dose escalation is typically limited by its anticholinergic side effects [84,85].

Cardiac myosin ATPase inhibitors represent the first targeted molecular therapy approved for obstructive HCM. Mavacamten and aficamten are two drugs currently approved for clinical use in adults, and they both act as reversible allosteric myosin inhibitors. Over the last four years, they revolutionized the treatment of LVOTO in HCM. In the EXPLORER-HCM, a randomized, double-blind, placebo-controlled, phase 3 trial, mavacamten improved the symptoms (NYHA Class) and functional capacity (peak VO_2_) in symptomatic patients in NYHA class II–III and normal ejection fraction compared to placebo, reducing the LVOT gradient by 30 mmHg in ≈30% of the patients [86]. Additionally, mavacamten showed independent improvement in the degree of mitral regurgitation and prolonged positive myocardial remodeling and improved left atrial volume and function in patients in sinus rhythm. It also supported continued avoidance of septal reduction therapies (SRT) in patients with obstructive HCM and severe symptoms at ≈2.5 years from the beginning of the treatment with mavacamten [87,88,89,90,91].

In the REDWOOD-HCM, a phase II, randomized, placebo-controlled study, aficamten significantly decreased the LVOT gradients and NT-proBNP levels in adult patients with symptomatic obstructive HCM [92]. More recently, in the SEQUOIA-HCM, a phase 3, randomized, double-blind trial, it showed an improved peak VO2 compared to placebo [93].

Cardiac myosin ATPase inhibitors are currently included as second-line therapy in both the European and American guidelines, in case first-line therapy is ineffective or poorly tolerated (IIa, level A) [7,75]. However, an ongoing head-to-head phase 3 trial (MAPLE-HCM) has been very recently presented to evaluate the efficacy and safety of aficamten as first-line therapy compared to metoprolol in adult patients with symptomatic obstructive HCM [91].

Mavacamten and aficamten are currently under investigation in adolescents with symptomatic obstructive hypertrophic cardiomyopathy, respectively in a phase 3 and 2 clinical trial (NCT06253221, NCT06412666) (https://doi.org/10.1161/circ.150.suppl_1.4132223) (accessed on 26 March 2025)

The list of the main drugs used in the treatment of LVOT obstruction and their respective dosages in the pediatric and adult populations are reported in Table 1.

Third-line therapy, invasive septal reduction therapy

SRT encompasses surgical septal myectomy and alcohol septal ablation (ASA). The approach to septal reduction (surgical versus percutaneous) should be tailored to the patient, depending on the anatomic features of the LVOT and the mitral valve, as well as the need for the presence of additional surgical targets [7].

According to the European Guidelines for adults, SRT is recommended (I, B) in severely symptomatic patients (NYHA Class III-IV, exertional or unexplained recurrent syncope) but may also be considered (IIb) in less symptomatic patients (NYHA Class II) with significant SAM-related mitral regurgitation, atrial fibrillation, or moderate-to-severe left atrial dilatation in expert centers with low complication rates [22]. Interestingly, according to the American guidelines, SRT can be considered in patients with symptomatic obstructive HCM as an alternative to titration of medical therapy after shared decision-making (2b C) [75].

In children with severe LVOTO who remain symptomatic despite medical therapy, surgery is indicated to relieve symptoms and enhance long-term survival [1]. Due to insufficient medium- to long-term safety and efficacy data, the use of ASA in children is currently not recommended (ESC). Transaortic septal myectomy, or the modified Konno procedure for children under 5 years [due to a small aortic annulus], is recommended for pediatric patients who require surgical relief of LVOTO [7,75].

Pacing

Dual-chamber pacing, with the aim of optimizing atrioventricular conduction and reducing the LVOT gradient, as well as allowing the up-titration of beta-blockers or verapamil, can be an option in patients with obstructive HCM. The European guidelines suggest considering dual-chamber pacing (IIb, level C) in patients who are symptomatic despite medical therapy and who are not candidates for SRT or who remain symptomatic despite medical therapy and are candidates for implantable cardioverter defibrillator (ICD) implantation (IIB, level C) [7], while the American guidelines seem more favorable (IIa, level B) to sequential pacing in patients with symptomatic obstructive HCM and who are candidates for ICD, especially if they are older than 65 years [75].

### 6.2. Atrial Fibrillation

Anticoagulation

Both European and American guidelines recommend anticoagulation in all adult patients with HCM and AF regardless of the CHA2DS2-VASc score (I, B), the American guidelines favoring it also in the case of subclinical/paroxysmal AF (recommendation I, level C for episodes longer than 24 h; IIa, C for episodes lasting between 5 min and 24 h). AF is uncommon in children with HCM, and there is a lack of data on the application of the CHA2DS2-VASc score or any other risk stratification tools. Additionally, the risks and benefits of prescribing oral anticoagulation in these cases have not been well studied [7].

Rhythm control

A rhythm control strategy in patients with HCM and AF is preferable when feasible [94]. Sinus rhythm restoration can be achieved via either electrical or pharmacological cardioversion. Catheter ablation is recommended in the European guidelines for patients who have failed or are intolerant to AAD or who have a high probability of tachycardia-induced cardiomyopathy as co-factors (I, level B), but it also should be considered, according to both European and American guidelines, as first-line therapy in selected patients or in patients with heart failure (IIa, level B).

Rate control

In the case of patients with HCM and AF in which a rate control strategy has been warranted, beta blockers, verapamil, or diltiazem can be used, according to the patient’s characteristics and comorbidities. Digoxin could also potentially be used in patients with HCM and without LVOTO [7,75].

### 6.3. Heart Failure and Chest Pain

HF should be managed according to the HF guidelines [7,95,96]. In the most recent European guidelines, the treatment of HF in HCM is encompassed in the general management of HF in cardiomyopathies, while the American guidelines specifically recommend discontinuing myosin inhibitors [I, level B] and negative inotropic agents (IIa, level C) and considering ICD implantation (IIa, level C) in patients with LV ejection fraction (EF) < 50% and minimizing the use of diuretics in patients with LVOTO [75].

Although beta-blockers and non-dihydropyridine calcium antagonists can be used in angina-like chest pain without LVOTO as first-line, the strength of recommendation differs according to the guidelines (I, level C in the American guidelines versus IIa, level C in the European guidelines). Moreover, the European guidelines suggest that oral nitrates and ranolazine may be considered as second-line agents [IIb, level C], while the American guidelines do not; however, they suggest apical myectomy in selected cases and if performed in dedicated centers (IIb, level C) [7,75].

### 6.4. Prevention of Sudden Cardiac Death

Implantation of a subcutaneous or endocavitary ICD is recommended in secondary prevention in patients who survived a cardiac arrest or a hemodynamically significant ventricular arrhythmia by both European and American guidelines (I, B), although only the ESC guidelines mention the life expectancy ≥ 1 year [7,75]. In the primary prevention of SCD, the approach of the European and American guidelines is slightly different, although both emphasize the need for multidisciplinary discussion and an ultimately patient-based decision, particularly in children, in view of the long-term device-related complications that may arise after the ICD implantation.

According to the European guidelines, ICD implantation should be considered in patients with HCM and a 5-year risk of death ≥ 6%, calculated with the HCM Risk-SCD calculator in patients aged ≥16 years [35] or with the HCM Risk-Kids calculator for children and adolescents < 16 years [IIa, level B] [33]. The recommendation is less strong in patients at intermediate risk or in patients at low risk of SDC and at least one clinical risk factor for SCD, including extensive LGE (>15%) at CMR or LVEF < 50% (IIb, level B) [7]. The American guidelines do not rely on risk-prediction scores, being more risk factor-oriented and considering ICD implantation in adults and in children with HCM and ≥ 1 risk factor for SCD (IIa, level B). According to the American guidelines, ICD implantation may also be considered (Class 2b, level B) in adult patients with HCM and extensive LGE at CMR (similarly to the ESC Guideline) or with documented NSVT [75].

**Scheme 2:**
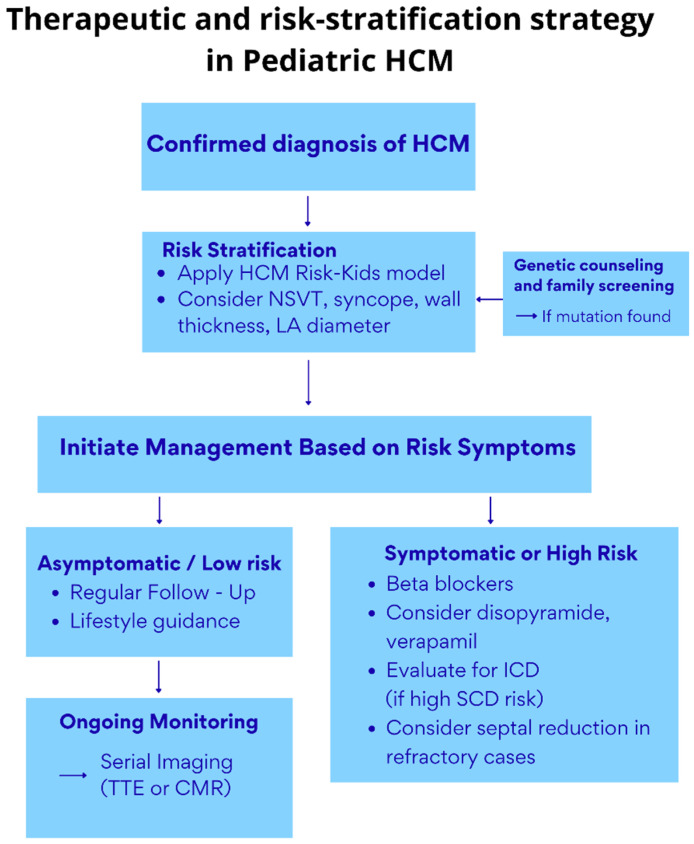
(HCM) hypertrophic cardiomyopathy; (NSVT) non-sustained ventricular tachycardia; (LA) left atrium; (ICD) Implantable Cardioverter Defibrillator; (SCD) sudden cardiac death; (TTE) transthoracic echocardiography; (CMR) cardiac magnetic resonance.

Scheme 2 summarizes the therapeutic and risk-stratification approach to MH in young patients.

## 7. Recommendations for Sports

Recent scientific advancements have led to a paradigm shift in the management of physical activity among pediatric patients with hypertrophic cardiomyopathy. Previously restrictive recommendations are being reconsidered in favor of a more integrative and individualized approach, emphasizing the role of structured physical activity, including pediatric cardiac rehabilitation programs, in improving the quality of life, psychological well-being, and functional capacity [97,98].

The most recent guidelines by the American Heart Association (AHA) and the American College of Cardiology (ACC), supported by contemporary evidence, suggest that, following comprehensive and individualized risk stratification, moderate to vigorous aerobic exercise may be safe and potentially beneficial for the majority of children and adolescents with HCM [98,99].

Moreover, participation in competitive sports is no longer contraindicated. Instead, it is increasingly evaluated on a case-by-case basis through a personalized approach led by a multidisciplinary team with expertise in pediatric cardiomyopathies. This process actively involves the patient and their family and places significant emphasis on the use of advanced diagnostic tools, such as cardiopulmonary exercise testing, cardiac magnetic resonance imaging, and ambulatory rhythm monitoring, which are essential for accurate and tailored risk assessment [99].

## 8. Future Directions

Recent findings from a multicenter retrospective study showed that children with RASopathies and severe HCM treated with a MAPK kinase (MEK) inhibitor (trametinib) with compassionate/off-label use had significantly reduced morbidity and mortality and improved clinical status compared to those treated with standard care, with manageable side effects [100]. In addition, recent data from 6420 childhood cancer survivors showed that common variants of TTN and BAG3 were associated with a reduced risk of late-onset cardiomyopathy [101].

These findings suggest that the genetic architecture of pediatric cardiomyopathies—whether dilated or hypertrophic—may differ substantially from adult-onset or familial forms.

Also, they support the concept of disease-specific and context-dependent genetic risk profiles, reinforcing the need for personalized approaches to risk stratification and surveillance in children at risk of cardiomyopathy.

## 9. Conclusions

Myocardial hypertrophy is a complex and multifaceted condition, requiring age-specific considerations and individualized management strategies. From prenatal life to adolescence, MH encompasses diverse etiologies, ranging from transient adaptations to progressive genetic cardiomyopathies and rare metabolic disorders.

Advances in diagnostic imaging, particularly echocardiography and cardiac magnetic resonance imaging, alongside enhanced molecular diagnostics, have significantly improved the capacity to accurately diagnose and characterize these conditions.

At the same time, this is particularly relevant, given the advances in therapeutic management and targeted therapies that significantly impact the prognosis and quality of life of these patients. Future research efforts should focus on refining the risk assessment tools, expanding our understanding of genotype–phenotype correlations, and developing further therapeutic approaches, including gene therapy.

## Figures and Tables

**Table 1:** Most frequently used medications for HCM with LVOT obstruction.

Classes	Drugs	Dosages (Children)	Dosages (Adults)
Non-vasodilating beta-blocker	Atenolol	0.5–1 mg/kg every 12–24 h (max 25–50 mg)	25–100 mg once daily
	Metoprolol	1–2 mg/kg every 6–12 h	25–100 mg twice daily
	Bisoprolol	0.2–0.4 mg/kg daily	1.25–20 mg once daily
	Propranolol	0.2–0.5 mg/kg every 6–8 h	10–25 mg every 6–8 h
Non-dihydropyridine calcium channel blockers	Verapamil	1–3 mg/kg every 8–12 h	40 mg every 12 h to 480 mg (extended release) once daily
	Diltiazem	1 mg/kg every 8 h	60 mg every 8 h to 360 mg (extended release) once daily
Class Ia antiarrhythmic drug	Disopyramide	1.5–4 mg/kg/dose every 6 h	400–600 mg/day
Cardiac myosin ATPase inhibitors	Mavacamten	Not available	Initial dose 5 mg once daily. Maximal dose 15 mg once daily
	Aficamten	Not available	Initial dose 5 mg once daily. Maximal dose 20 mg once daily

## Abbreviations

ADD	Antiarrhythmic drug
AF	Atrial fibrillation
ASA	Alcohol septal ablation
AS	Aortic stenosis
CHD	Congenital heart disease
CMR	Cardiac Magnetic Resonance
CoA	Coarctation of the aorta
CT	Computed tomography
DCM	Dilated cardiomyopathy
EF	Ejection Fraction
EORP	EURObservational Research Programme
GLS	Global longitudinal strain
HCM	Hypertrophic cardiomyopathy
HF	Heart Failure
ICD	Implantable Cardioverter Defibrillator
IEM	Inborn errors of metabolism
LGE	Late gadolinium enhancement
LV	Left ventricle
LVH	Left ventricular hypertrophy
LVOT	Left ventricle outflow tract
LVOTO	Left ventricle outflow tract obstruction
LVNC	Noncompacted myocardium
MH	Myocardial hypertrophy
NSVT	Non-sustained ventricular tachycardia
RCM	Restrictive cardiomyopathy
RV	Right ventricle
SAM	Systolic anterior motion
SRT	Septal reduction therapies
STE	Speckle-tracking echocardiography
SCD	Sudden cardiac death
TTE	Transthoracic echocardiography
TTTS	Twin–twin transfusion syndrome
